# Macrophages, Chronic Inflammation, and Insulin Resistance

**DOI:** 10.3390/cells11193001

**Published:** 2022-09-26

**Authors:** He Li, Ya Meng, Shuwang He, Xiaochuan Tan, Yujia Zhang, Xiuli Zhang, Lulu Wang, Wensheng Zheng

**Affiliations:** 1 Beijing City Key Laboratory of Drug Delivery Technology and Novel Formulation, Institute of Materia Medica, Chinese Academy of Medical Sciences & Peking Union Medical College, Beijing 100050, China; 2 Shandong DYNE Marine Biopharmaceutical Co., Ltd., Rongcheng 264300, China; 3 Institute of Medicinal Biotechnology, Chinese Academy of Medical Science and Peking Union Medical College, Beijing 100050, China

**Keywords:** obesity, macrophages, chronic inflammation, insulin resistance, molecular mechanism

## Abstract

The prevalence of obesity has reached alarming levels, which is considered a major risk factor for several metabolic diseases, including type 2 diabetes (T2D), non-alcoholic fatty liver, atherosclerosis, and ischemic cardiovascular disease. Obesity-induced chronic, low-grade inflammation may lead to insulin resistance, and it is well-recognized that macrophages play a major role in such inflammation. In the current review, the molecular mechanisms underlying macrophages, low-grade tissue inflammation, insulin resistance, and T2D are described. Also, the role of macrophages in obesity-induced insulin resistance is presented, and therapeutic drugs and recent advances targeting macrophages for the treatment of T2D are introduced.

## 1. Introduction

The incidence of obesity is widespread in both developing and developed countries [1]. It is estimated that 57.8% of the global adult population will be overweight or obese by 2030 [2]. Obesity does not only affect adults, as the incidence of overweight and obesity in children has also risen sharply in recent years [3]. Obesity has a significant impact on the physiological functions of the human body and increases the risk of many metabolic diseases, including type 2 diabetes (T2D) [4,5], non-alcoholic fatty liver [6,7,8], atherosclerosis [9,10], cardiovascular disease [5], musculoskeletal diseases [11], and cancer [12,13,14]. This affects the quality of life of these patients and increases medical care costs.

Among them, obesity is closely related to the development of insulin resistance. Insulin target organs, such as adipose tissue, skeletal muscle, and liver, are affected by obesity at the morphological, functional, and molecular levels [15]. As one of the causes of T2D [16,17], insulin resistance is a pathological condition in which cells fail to respond normally to insulin stimulation [18]. Obesity causes various changes in adipose tissue, including metabolic and endocrine functions, such as the increased release of fatty acids, hormones, and proinflammatory molecules [19]. Lipid accumulation in skeletal muscle is associated with insulin resistance and markedly impairs glucose disposal function of skeletal muscle [20,21,22,23]. Insulin resistance induced by obesity in the liver characterized by impairment in the ability of insulin to inhibit glucose output, finally resulting in gluconeogenesis [24]. In response to high blood glucose levels, β-cells promote insulin production and lead to hyperinsulinemia [25]. Therefore, insulin resistance is often accompanied by hyperglycemia and hyperinsulinemia [26], until β-cells cannot maintain a compensatory increase in insulin, eventually leading to T2D [27].

Many molecular mechanisms have been proposed between obesity, insulin resistance, and T2D, including endoplasmic reticulum stress, oxidative stress, lipid homeostasis dysregulation, mitochondrial dysfunction, and hypoxia [28], but the molecular link is not fully understood. This review focuses on the chronic inflammation induced by obesity as a possible contributor to insulin resistance [29]. In obesity, changes in the polarization state of macrophages in the adipose tissue, liver, and muscle were detected [30], and the interactions between macrophages and insulin target organs may influence both metabolism and inflammation [31].

This review focuses on the molecular mechanisms between macrophages, chronic inflammation, insulin resistance, and T2D. We discuss the polarization, distribution, and accumulation of macrophages in insulin target organs such as adipose tissue, skeletal muscle, and liver, and summarize the relationship between macrophages and insulin resistance, as well as therapeutic drugs and recent advances targeting macrophages for the treatment of T2D.

## 2. Classification of Macrophages

Macrophages are a type of phagocyte and antigen-presenting cell, which can secrete trophic factors, immune mediators, and effectors, phagocytose pathogens, or cell debris, as well as process antigen and present antigen on the cell surface [32,33]. Diversity and plasticity are hallmarks of macrophages [34]. Two recognized subtypes of macrophages are classically activated M1 macrophages and alternately activated M2 macrophages [35,36] that represent two extremes of a dynamic changing state of macrophage activation [37]. M1 macrophages could be induced by IFN-γ, TNF-α, GM-CSF, and lipopolysaccharide (LPS) [38], while M2 macrophages are stimulated with IL-4 or IL-13 [37,39]. M1 macrophages are involved in promoting Th1 response, possessing strong microbicidal and tumoricidal activity mainly by the secretion of cytokines that inhibit the proliferation and damage of contiguous tissue, including TNF-α, IL6, IL-12, IL-23, nitric oxide, reactive oxygen, and inducible nitric oxide synthase (iNOS) [40,41]. M2 macrophages release arginase-1, IL-10, ornithine, and polyamines, and promote Th2 response, proliferation, tissue repair, immune tolerance, and tumor progression [42,43,44,45]. The M2 macrophages can be divided into four subdivisions based on the stimuli and induced transcriptional changes: alternatively activated macrophages activated by IL-4 or IL-13 (M2a), type 2 macrophages stimulated by immune complexes and LPS (M2b), deactivated macrophages activated by glucocorticoids or IL-10 (M2c), and M2-like macrophages activated by adenosines or IL-6 (M2d) [46,47]. M2 macrophages use oxidative metabolism to fulfill their functions, while M1 macrophages obtain energy through glycolysis, which favors the production of the proinflammatory cytokine IL-1β and nitric oxide [48]. 

It has also been pointed out that the use of M1 and M2 to classify macrophages is too polarized; the phenotype of macrophages is determined by many stimulating factors and cannot be generalized by limited subtypes [34]. New subtypes of macrophages different from M1 and M2 are constantly being discovered. Jaitin et al. found a new Trem2+ lipid-associated macrophage (LAM) subset after high-fat diet (HFD), which is the most strongly expanded immune cell subset of adipose tissue in the state of obesity. Trem2+ LAMs facilitate the processing and degradation of lipids by highly expressing fatty acids transporters Cd36, fatty acid binding proteins 4 and 5 (Fabp4, Fabp5), lipoprotein lipase (Lpl) and lysosomal acid lipase (Lipa). Jaitin et al. also discovered that Trem2 expression is an important factor in the prevention of adipose tissue dysfunction and metabolic disorders in obesity and that loss of Trem2 aggravates WAT hypertrophy in response to HFD feeding [49]. Moreover, Trem2+ macrophages are found in the plaque of mice with both early and advanced atherosclerotic lesions [50]. One study reported that Trem2+ macrophages express high levels of Trem2, Cd9, Spp1, and cathepsins (Ctsb, Ctsd and Ctsz), which is associated with lipid metabolic processes and lesion calcification while downregulating pro-inflammatory genes, and further finding that macrophage Trem2 negatively correlates with plaque stability [51].

Instead of fixed states, M1/M2 polarization is a dynamic process that can be reversed under physiological and pathological conditions [52]. A variety of signaling molecules and transcription factors are involved in regulating the polarization of macrophages, including PPAR, KLF, IRF, STAT, NF-κB, and HIF families. IRF/STAT signaling is a major pathway in modulating macrophage polarization. IFN-γ and LPS stimulated IRF/STAT signaling activates M1 phenotype via STAT1, IL-4, and IL-13, while M2 phenotype is activated via STAT6 [53]. It is also influenced by local microenvironmental conditions such as hypoxia [54] and diseases such as obesity, which are discussed in this article.

Macrophages can also be classified based on their locations and functions. Tissue-resident macrophages are different from monocytes that come from the bone marrow and circulate in the blood [55]. Different tissues have specialized tissue-resident macrophages, including Kupffer cells in the liver, adipose tissue macrophages (ATMs) in the adipose tissue, alveolar macrophages in the lung, red pulp and marginal zone macrophages in the spleen, and microglia in the brain. Kupffer cells and ATMs are the two major metabolic tissue macrophages [56]. ATMs in murine have been characterized as F4/80^+^, CD11b^+^, CD206^+^, and CD301^+^ cells, while human ATMs are characteristically CD14^+^/CD16^−^ and express markers CD68, CD163, CD204, and CD206. It has been established that ATMs in humans barely express markers for M1 and M2 classification, such as iNOS and arginase-1 [57]. Further, the KCs in mice are typically characterized by F4/80^hi^, CD11b^int^, CD68^+^ cells. Additionally, T-cell immunoglobulin, mucin domain containing 4 (Tim4), and C-type lectin domain family 4 member F (Clec4F) have been described as surface markers specific to KCs [58,59]. The murine monocytes infiltrating the liver, on the other hand, are characterized as CD11b^+^, Cx3cr1^+^, Ly6c^+^, CCR2. When KCs are depleted or tissues are injured or inflamed, monocytes become significant contributors to the liver macrophage pool [59]. KCs in humans lack distinctive markers and have often been characterized by their expression of CD68 and CD14. Recently study have shown that humans KCs also express MARCO, CD163, and Tim4 [60]. There is less understanding of the markers for monocytes in the human liver, but include CCR2, Cx3cr1, SA100A2, and CD14 [60].

## 3. Macrophage- and Obesity-Induced Insulin Resistance

Macrophages in insulin target the organs, including adipose tissue, liver, and muscle, that play a central role in inflammation and insulin resistance [61]. In the obese state, macrophages infiltrate the target organ, are activated to the M1 polarization state and produce abundant inflammatory cytokines, which negatively affect the transmission of insulin signals and increase the development of chronic inflammation, as well as insulin resistance [62,63,64].

### 3.1. Adipose Tissue and ATMs

Adipose tissue is a reservoir of fatty acids during fasting. When glucose levels have returned to normal after meals, free fatty acids (FFAs) are released into the circulation by adipose tissue and used by other tissues as an energy source [65]. They are also the main site of inflammation in the obese state, and the accumulation of ATMs is the key factor in modulating inflammation [66]. Compared to subcutaneous adipose tissue, VAT plays a critical role in insulin resistance, and the content of ATMs in VAT is also higher than that of subcutaneous adipose tissue [67]. The ATM pool in lean mice originates from yolk-sac progenitors and self-renews by proliferation [68,69]. Eventually, these resident ATMs are replaced by bone-marrow-derived macrophages, likely from monocytes [70]. Three major ATM populations have been described in lean mice. Primitive adipose tissue is populated by LYVE1^+^ ATMs, which are closely associated with vasculature [71,72]. Further investigations identified one or two CD63^+^ monocyte-derived ATM subpopulations based on differential expression of MHCII, CD11c, and CX3CR1 [49,73,74]. The separation of the two monocyte-derived ATMs remains unclear, and CD11c expression can be attributed to either.

ATMs are mainly in the M2 polarization state in the lean state [75]. Preventing the ability of macrophages from transitioning to the M2 state leads to accelerated weight gain and glucose intolerance in mice [76,77]. M2 macrophages can maintain insulin sensitivity and glucose homeostasis by secreting factors such as IL-10, IL-1, and catecholamines to regulate lipid metabolism, block inflammation response, and increase insulin sensitivity [78,79,80]. Although conflicting evidence suggests that M2 macrophages do not contribute to adipocyte metabolism or adaptive thermogenesis via the production of catecholamines [81], evidence also shows that macrophages in adipose tissue are capable of taking up and degrading catecholamines released by neurons and that this system could be improved by obesity and aging, resulting in a decreased response to cold stress and starvation [82,83].

One of the hallmarks of obesity-related chronic inflammation is the accumulation of ATMs [19]. Macrophages account for approximately 10% of white adipose tissue (WAT) in lean mice and humans compared to nearly 40% in obese humans and over 50% in extremely obese, leptin-deficient mice [19,84]. Preventing the accumulation of ATMs during obesity can inhibit the development of inflammation and insulin resistance [85]. Past studies believed that these macrophages were derived from peripheral blood mononuclear cells. However, recent studies have shown that the significant increase in ATMs in the early stage of obesity is mainly due to in situ macrophage proliferation, and migrating monocytes favor the accumulating of ATMs in the relatively late stages of obesity [86]. ATMs in obese mice are mainly in the M1 state, which can produce a large number of proinflammatory cytokines to influence the chronic inflammation state. The mechanism of obesity leading to changes in the polarization status of ATMs has not been fully explained, and its influencing factors may be multiple. Lumeng et al. had found that the M1 macrophages present in the inflammatory aggregates of adipose tissue originate from circulating monocytes, instead of being directly converted from M2 to M1 polarization state [87]. Expansion of adipose tissue may lead to adipocyte hypertrophy and hyperplasia, then releasing signal molecules such as MCP-1 and other inflammatory cytokines and chemokines, attracting a large number of bone marrow-derived monocytes to enter adipose tissue and subsequently differentiate into macrophages [19,88]. In addition, the chronic inflammation induced by obesity not only leads to the accumulation of macrophages, but also impairs the macrophage egress [89].

There are also differences in the distribution of macrophages in adipose tissue between lean and obese states: macrophages are evenly distributed throughout the adipose tissue in the lean state; while in the obese state, macrophages mainly appear in ring-like structures around dying adipocytes, known as crown-like structures (CLSs), to phagocytose the cells and continuously release proinflammatory cytokines [90]. Dying adipocytes display engulfment signals, inducing phagocytic cells to migrate to the site of cell death [85]. HFD-feeding increases the number of CLSs and the expression of proinflammatory cytokines [88]. Furthermore, Hill et al. found that obese adipose tissue contains multiple distinct ATMs populations, with Ly6c+ ATMs located outside the CLSs and are adipogenic. By inducing genes involved in cholesterol and lipid biosynthesis, Ly6c ATMs restore normal adipose physiology after adoptive transfer. The lipid-rich CD9+ ATMs located within the CLSs are responsible for the inflammatory signature of obese adipose tissue. In lean mice, adoptive transfer of CD9+ ATMs induces obesity-associated inflammation [73]. In contrast to CD9− ATMs, CD9+ ATMs express higher levels of CD16 and CD206 and are enriched for transcription factors AP-1 and NF-κB with associated genes such as Ccl2, Il1a, Il18, and Tnf [73]. The aforementioned Trem2+ lipid-associated macrophages are also recognized as the main expanded immune cell subset in adipose tissue during obesity [49].

### 3.2. Liver and Kupffer Cells

The liver is the largest solid organ in the body [91]. It plays an indispensable role in neutralizing and eliminating toxic substances and regulating metabolism [92,93]. In response to postprandial hyperglycemia, the liver converts excess glucose into glycogen, and during fasting, the liver maintains blood glucose levels through glucose production [93]. Among all organs, the liver has the largest proportion of macrophages, with about 20–35% of hepatic non-parenchymal cells and 80–90% of tissue macrophages in the host [94].

The two macrophage populations in the liver include Kupffer cells and monocyte-derived macrophages. During obesity, Kupffer cells are activated to the M1 state, which attracts inflammatory monocytes into the liver and promotes their differentiation into monocyte-derived macrophages [95,96], resulting in an increased proportion of macrophages in the liver [97]. Monocyte-derived macrophages and inflammatory-activated Kupffer cells contribute to obesity-induced hepatic inflammation and insulin resistance. On the other side, the anti-inflammatory activation of Kupffer cells could reduce obesity-induced insulin resistance [98]. Monocyte-derived macrophages are smaller than Kupffer cells in mice [99], and the number of monocyte-derived macrophages is six-fold higher in HFD feeding mice compared to lean mice, while the number of Kupffer cells is similar between the two groups [100]. Correspondingly, Kupffer cells and monocyte-derived macrophages are essential for maintaining the homeostasis of the liver and whole body [101].

Chronic inflammation is known to induce insulin resistance in the liver [102,103]. Kupffer cells play a major role in liver insulin resistance induced by HFD feeding through producing various inflammatory mediators, including TNF-α, IL-6, IL-1β, prostaglandins, and reactive oxygen species, which play vital roles in promoting the development of insulin resistance [104,105,106,107,108]. Intravenous infusion of clodronate can selectively deplete Kupffer cells without affecting ATMs, which is found to enhance insulin signaling and hepatic insulin sensitivity significantly in the case of an HFD; it could also reduce adipose tissue weight without affecting body weight, liver weight, or steatosis [104]. After treatment with GdCl_3_, a specific inhibitor of Kupffer cells, phagocytosis, and other functions of Kupffer cells were inhibited, and whole-body glucose tolerance was increased in mice [107]. Moreover, lipid accumulation in the liver caused by increased fatty acid absorption, increased liver synthesis, or reduced fatty acid oxidation in obesity can be used to predict T2D [103,109], and lipid metabolisms induced by macrophages-derived cytokines lead to lipotoxicity, which in turn causes liver inflammation and insulin resistance [110].

### 3.3. Muscle and Macrophages

Muscle mass is an important determinant of glucose tolerance, as less muscle mass has been associated with greater insulin resistance [111,112]. Increased blood glucose levels after meals induce insulin secretion, in which case the skeletal muscle is stimulated to absorb glucose, and approximately 60% of ingested glucose is taken up by skeletal muscle [113,114,115]. Insulin resistance in skeletal muscle is manifested by a decrease in insulin-stimulated glucose uptake, associated with impaired insulin signaling, glucose transport, oxidative phosphorylation, and glycogen synthesis [22].

**Figure 1:**
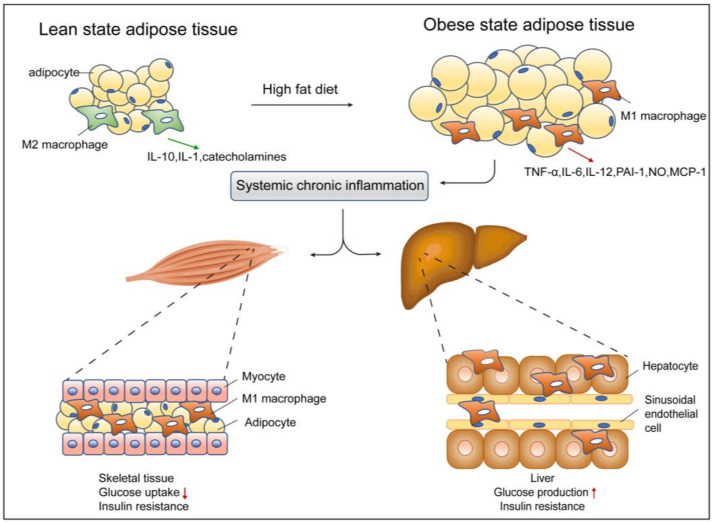
Obesity induces macrophage infiltration and insulin resistance in adipose tissue, liver, and skeletal muscle. In the lean state, tissue-resident M2 macrophages in adipose tissue secrete anti-inflammatory factors such as IL-1 and IL-10 to maintain an insulin-sensitive environment. During obesity, increased levels of nutrients result in adipocyte hypertrophy and apoptosis. Proinflammatory mediators result in M2 macrophage polarization into the M1 state, which triggers adipose tissue chronic inflammation. Other metabolic tissues, including skeletal muscle and liver, are also influenced by increased cytokine production and macrophage recruitment, resulting in lower glucose uptake in skeletal muscle and higher glucose production in the liver, fueling the insulin resistance state further.

Insulin resistance in skeletal muscle is caused by impaired insulin signaling and many post-receptor intracellular defects including impaired glucose transport, glucose phosphorylation, and reduced glucose oxidation and glycogen synthesis. Macrophage recruitment into skeletal muscle tissue is also involved in promoting insulin resistance [23,85,116]. Macrophages in skeletal muscle display phenotypic diversity and can switch between an M1 and M2 phenotype in response to changes in the specific environment. For example, macrophages are polarized to M1 phenotype in response to circulating FFAs, leading to release of pro-inflammatory cytokines [117]. Macrophage content in skeletal muscle correlates negatively and dynamically with insulin sensitivity [117,118]. The number of CD68^+^ macrophages in the skeletal muscle of obese non-diabetic subjects is increased compared to lean subjects [117]. Studies have also found that HFD increases the CD11b^+^ F4/80^+^ macrophage numbers in muscle compared to lean mice [19,119]. During obesity, macrophages alter the inflammatory state of muscle cells by secreting cytokines and chemokines [117]. The chronic inflammatory condition impairs lipogenesis and lipolysis of adipose tissue, resulting in increased circulating FFAs and triggering ectopic fat deposition in skeletal muscle [114,120], and inflammatory cytokines secreted from macrophages induce lipolysis and lead to increased levels of lipid metabolisms, which is highly correlated with skeletal muscle insulin resistance [121] (Figure 1).

### 3.4. Pancreas and Macrophages

Macrophages are constitutively present within islets under normal physiological conditions, where they play important roles in pancreas development and pancreas homeostasis. Macrophages are present during mouse embryonic development. In op/op mice lacking macrophages in the pancreas, it was found that these mice had less β-cell mass than normal mice during embryonic development and after birth, indicating that pancreas macrophages are necessary for increasing β-cell mass [122]. The mechanism by which macrophages support pancreas development is unclear, but may be related to cytokine production by macrophages, as low concentrations of IL-1β can stimulate β-cell proliferation [123]. Islet-resident macrophages also exert protective effects and contribute to the maintenance of islet homeostasis. Islet-resident macrophages express arginase I, CD206, and IL-10, etc., which are similar to M2 macrophages.

Numerous studies have revealed that macrophage infiltration in islets is increased in T2DM, C57BL/6 mice fed HFD, and db/db mice [124], and islet-resident macrophages content generally correlates with the degree of β-cell dysfunction [124,125]. High glucose could induce the secretion of chemokines from islets, which promoted monocyte and neutrophil migration. It is possible that the T2D milieu may induce chemokine production and promote macrophage infiltration into pancreas. Islets contain two subpopulations of macrophages: CD11b^+^Ly^-^6C^+^ monocytes/macrophages and CD11b^+^Ly^-^6C^−^ macrophages [126]. Islet-resident macrophages were mostly CD11b^+^Ly^-^6C^−^ cells, which exhibit an M2 phenotype in basal conditions. Compared to control db/+ and KKTa mice, fractions of these M2-type cells were not altered in db/db or KKAy mice. However, the number of CD11b^+^Ly^-^6C^+^ macrophages was significantly increased in the T2D models. These cells express inflammatory cytokines, such as IL-1β and TNF-α, and exhibit an M1-type phenotype, which may indicate that macrophage polarity appears to be shifted towards M1 in T2D islets.

## 4. Mechanism of Inflammation and Insulin Resistance

Several inflammatory signaling pathways are implicated in inhibiting insulin functions, which can link inflammation to insulin resistance. JNK, IKK/NF-κB, JAK/STAT, and other pathways play major roles in forming chronic inflammation [127,128,129,130,131]. Inflammatory signaling is activated by several mechanisms, including the release of endogenous factors such as saturated fatty acids and heat shock proteins known as danger- or damage-associated molecular patterns (DAMPs), which are recognized by pattern recognition receptors (PRRs), and activate inflammation processes as well as inflammatory factors in the absence of exogenous factors such as pathogens [132]. DAMPs are produced largely by cellular stress, tissue damage, and inflammation [133]. PRRs include several types, such as Toll-like receptors (TLRs), RIG-I-like RNA helicases (RLHs), NOD-like receptors (NLRs), and AIM2-like receptors (ALRs) [134]. The PRRs can also recognize pathogen-associated molecular patterns (PAMPs) on pathogens and biological allergens, including LPS, peptidoglycan, and bacterial DNA [135,136].

### 4.1. JNK Pathway

Hotamisligil et al. found that the mRNA and protein levels of TNF-α in adipose tissue are increased under insulin resistance and obesity, and insulin sensitivity is improved after neutralizing TNF-α [66,137]. Further studies found that TNF-α is an adipose tissue-derived proinflammatory cytokine that is involved in obesity-induced insulin resistance [19]. Helped by TNFR-1, TNF-α plays an important role in activating and recruiting the immune cells to propagate inflammation. At that time, TNF-α was recognized to derive from adipocytes in obesity, and Xu et al. found that the WAT stromal vascular fraction (SVF) also secretes TNF-α [138]. Moreover, Taeye et al. revealed that macrophages are the main sources of TNF-α in the obese WAT [139], transplanted TNF-α deficient mice with TNF-α-sufficient or TNF-α-deficient bone marrow and found that macrophage-derived TNF-α contributes to inflammation development and insulin resistance in diet-induced obesity [139]. TNF-α knockout obese mice have improved insulin sensitivity compared to the control group [140]. The studies further found downstream signaling at the TNF-α-induced kinase levels. TNF-α induces a dual kinase system, including JNK kinases and IKK complex [141]. In the case of insulin resistance, JNK-1 and IKK signals are upregulated in adipose tissue [138], skeletal muscle [142], and liver [143]. JNK have JNK-1 and JNK-2 subsets, and JNK-1 plays a key role in the development of insulin resistance in adipose tissue and muscle [144]. JNK can be activated by TNF-α, IL-1β, and ultraviolet light [145]. JNK pathway is involved in impaired insulin signaling by causing the serine phosphorylation of insulin receptor substrate 1 (IRS-1), which reduces the tyrosine phosphorylation of IRS-1, thereby reducing PI3K and AKT signaling [146,147].

New research has found that proinflammatory molecule leukotriene B4 (LTB4) also induces insulin resistance through the JNK pathway [148]. LTB4 is a neutrophil chemotactic agent produced by arachidonic acid metabolism [149,150]. It is secreted by both immune cells, such as macrophages and eosinophils, and primary tissue cells, such as adipocytes, hepatocytes, myocytes, and endothelial cells [148]. LTB4 levels increase and play key roles in obesity-induced insulin resistance through the G protein-coupled receptor BLT-1, which is distributed mainly on immune cells and primary metabolite cells [148]. When primary tissue cells produce LTB4, it causes macrophages chemotaxis and migration into tissues, and macrophages secrete more LTB4 and act on primary tissue cells in a positive feedback loop, further inducing chronic tissue inflammation [148]. LTB4 treatment could stimulate JNK activity and enhance IRS-1 serine phosphorylation, impairing insulin sensitivity in target cells [148]. Treatment of adipocytes and hepatocytes with LTB4 in vitro induces insulin resistance, and knockout of BLT-1 can improve adipose tissue inflammation and insulin sensitivity in obese mice [148].

### 4.2. IKK/NF-κB Pathway

In addition to the JNK signaling pathway, insulin resistance is also closely related to NF-κB activation, which is induced by TNF-α [116]. NF-κB is a transcription factor of Rel family proteins and is involved in inflammation and immune responses. IκB retains NF-κB in an inhibitory cytoplasmic complex at a steady-state, and IKK phosphorylation could phosphorylate IκBα under inflammation, which separates and degrades IκBα from NF-κB [151], allowing free NF-κB to translocate to the nucleus and interact with related DNA response elements binding, which induces transactivation of inflammatory genes such as TNF-α, IL-1β, and IL-6, further contributing to insulin resistance [152]. IKK2 is a central coordinator of inflammatory responses to activate NF-κB. Yuan et al. first showed the importance of NF-κB signaling in metabolic disorders, in that IKK2 heterozygous-deletion mice have improved glucose tolerance and reduced basal glucose and insulin values in HFD feeding compared with control [153]. Chiang et al. found that HFD feeding increases NF-κB activation in mice, which leads to an increased level of IKKɛ in hepatocytes and adipocytes, and IKKɛ knockout mice are protected from HFD-induced obesity and chronic inflammation [154].

IL-1β participates in insulin resistance through NF-κB pathway. IL-1β belongs to the IL-1 superfamily and is produced mainly by monocytes and macrophages and regulated by inflammasome activity [155]. The levels of IL-1β are increased during hyperglycemia [156], and elevation of IL-1β has been shown to increase the risk of T2D [157]. IL-1β binds to interleukin-1 receptor type I (IL-1R1) and triggers intracellular signaling cascades. IL-1R1 activates JAK protein kinases, then stimulates the translocation of NF-κB to the nucleus from a complex with IκB, thus promoting inflammatory gene expression [158]. IL-1β impairs insulin signaling in peripheral tissues and reduces insulin sensitivity and secretion in β-cells [159]. Gao et al. observed that macrophage-conditioned (MC) medium significantly reduces protein abundance of insulin signaling molecules in human adipocytes and blocks the actions of IL-1β, which can reverse the effects of MC medium on insulin signaling [155]. Additionally, IL-1β depletion completely changes the inhibitory effect of MC medium on insulin signaling molecules like IRS-1 and PI3K, which indicates that IL-1β is a key factor in mediating macrophage-induced insulin resistance in adipocytes [155].

Moreover, monocytes and tissue-resident macrophages express high levels of PPAR-γ [160]. PPAR-γ is a member of the nuclear receptor superfamily and is involved in the expression of the inflammatory response, cell differentiation, lipid, and glucose metabolism genes [161]. PPAR-γ natural ligands include various lipid and prostaglandin (PGs), and PGs could suppress PPAR-γ functions [162]. Under inflammation, the production of PGs increases to a considerable extent, which has a positive correlation with insulin resistance [163]. In contrast, PPAR-γ synthetic agonists significantly reduce insulin resistance and are widely used to treat diabetes [164,165]. PPAR-γ suppresses the NF-κB transcription activity and inhibits inflammation by interacting with p65 to induce ubiquitination and degradation. PPAR-γ also upregulates the IRS protein, which improves insulin resistance induced by obesity [166,167]. Macrophages with PPAR-γ knockout lead to decreased systematic glucose tolerance, increased insulin resistance, and increased levels of inflammatory factors expression in the muscles and liver [168].

### 4.3. JAK/STAT Pathway

IL-6 plays a crucial role in regulating metabolism and immunity. It is also elevated in obese patients compared to lean controls, which is detrimental to metabolic balance [169]. IL-6 works by binding to IL-6 receptor α chain and GP130 signaling chain complex in classical membrane-bound pathway, then initiating the JAK2/STAT3-dependent transcriptional activation of target genes, including SOCS-3 [170]. SOCS-3 is a negative regulator of IL-6 signaling and could impair insulin signal transduction at IRS protein level. IL-6-induced SOCS-3 leads to proteasomal degradation of IRS-1 [171]. Studies of IL-6 functions on insulin sensitivity have shown conflicting results. A very-low-calorie diet and weight loss decrease IL-6 levels significantly in adipose tissue and serum and improve insulin sensitivity compared to the control [172]. Acute IL-6 infusion of mice leads to insulin resistance without obesity [173]. However, IL-6 deficient mice develop insulin resistance and mature-onset obesity [174]. Another study showed that 4-month-old IL-6 deficient mice have glucose intolerance and increased fat pad weight [175]. Additional investigation is required to speculate on the effect of IL-6 on insulin sensitivity.

### 4.4. Other Pathways

Cytokines derived from macrophages, including TNF-α, IL-6, and IL-1β, could influence lipolysis. TNF-α affects lipid metabolism by inducing lipolysis, inhibiting FFAs uptake and lipoprotein lipase activity [176]. Starnes et al. administered TNF-α to patients and found that FFAs metabolism increased >60% [177], and TNF-α-deficient obese mice have lower levels of FFAs [140]. IL-6 enhances lipolysis and fatty acid oxidation both in mice and humans [178]. Wolsk et al. found that the unidirectional release of muscle fatty acids during IL-6 acute infusion leads to an increase in systemic fatty acids, which indicates that IL-6 may be a direct regulator of fat metabolism in skeletal muscle [179]. Moreover, IL-1β indirectly stimulates lipolysis by reducing the production and activity of proteins that inhibit lipolysis and increase the release of FFAs and glycerin [180]. Increased levels of lipid metabolites, including FFAs, ceramides, and diacylglycerol (DAG), may cause dysfunction and insulin resistance [181]. High levels of FFAs are considered markers of insulin resistance and T2D [182,183]. Elevated FFAs inhibit pyruvate dehydrogenase, increase serine phosphorylation of IRS-1, impair insulin-stimulated glucose uptake, and decrease glucose oxidation [184]. Several studies have found a correlation between the accumulation of DAG and impaired insulin function [185,186]. DAGs activate the PKCθ and PKCδ subtypes of protein kinase C (PKC) [187], and PKC prevents insulin-stimulated tyrosine phosphorylation of IRS-1 and directly interferes with the insulin signaling pathway [186]. PKC-θ knockout mice are protected against fat-induced insulin signaling defects and systemic insulin resistance [188]. Ceramides are implicated as antagonists of insulin action, and plasma ceramides are at high levels in obese or diabetic individuals [189]. Ceramide is mainly involved in the development of insulin resistance from two aspects: ceramide signal stimulates the binding of PKCζ and AKT, making AKT unable to bind phosphatidylinositol (3,4,5) -triphosphate (PIP3), thereby inhibiting insulin signaling [190]; on the other hand, ceramide activates protein phosphatase 2A (PP2A) and phosphorylates it, thereby impairing AKT [191]. Ceramides decrease the plasma content during pioglitazone treatment, and inhibition of ceramide synthesis or stimulation of ceramide degradation improves insulin signaling, indicating indispensable participation of ceramides in the development of obesity and insulin resistance [189].

Furthermore, galectin-3 (gal-3) is a member of the galectin family that induces insulin resistance [192,193]. Gal-3 binds directly to the insulin receptor and inhibits downstream signaling [192]. The amount of gal-3 in the blood is higher in the obese state than in the lean state [192]. Treatment of adipocytes, hepatocytes, and myocytes with gal-3 in vitro induces insulin resistance [192]. Administration of gal-3 in vivo can also cause glucose intolerance and insulin resistance in mice, and gal-3 knockout mice have higher insulin sensitivity and glucose tolerance [192]. The above results suggested that gal-3 may be a critical molecule in insulin resistance.

**Figure 2:**
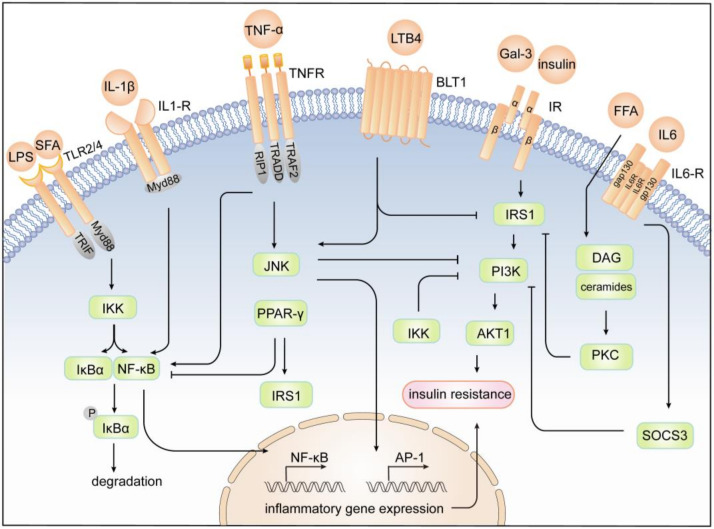
Cytokines regulate insulin sensitivity in insulin target cells. Activation of the insulin receptor leads to tyrosine phosphorylation of IRS-1 and initiates insulin signal transduction. Activation of TLR2/4 and TNFR results in the promotion of NF-κB and JNK signaling pathways. The serine kinases IKK and JNK-1 could reduce the signaling of IRS-1 and impair downstream insulin signaling. Moreover, the activation of IKK causes phosphorylation and degradation of IκB; thus, NF-κB could translocate into the nucleus. JNK promotes the formation of the AP-1 transcription factor, which in turn transactivates inflammatory gene expression by NF-κB and AP-1, further contributing to insulin resistance. PPAR-γ suppresses the NF-κB transcription activity and upregulates IRS protein, which favors the improvement insulin sensitivity. LTB4 promotes JNK activation through BLT-1 and leads to subsequent IRS-1 serine phosphorylation, ultimately promoting cellular insulin resistance. Gal-3 directly inhibits the insulin receptor and impairs all the major steps in the insulin signaling pathway. Additionally, IL6 induces SOCS-3 and leads to proteasomal degradation of IRS-1 through binding to the IL-6 receptor. IL-1β stimulates the translocation of NF-κB to the nucleus through IL-1R1, promoting inflammatory gene expression. Also, lipid metabolites, including FFAs, ceramides, and DAG, activate the PKC to impair IRS-1, which directly interfere with insulin signaling.

**Figure 3:**
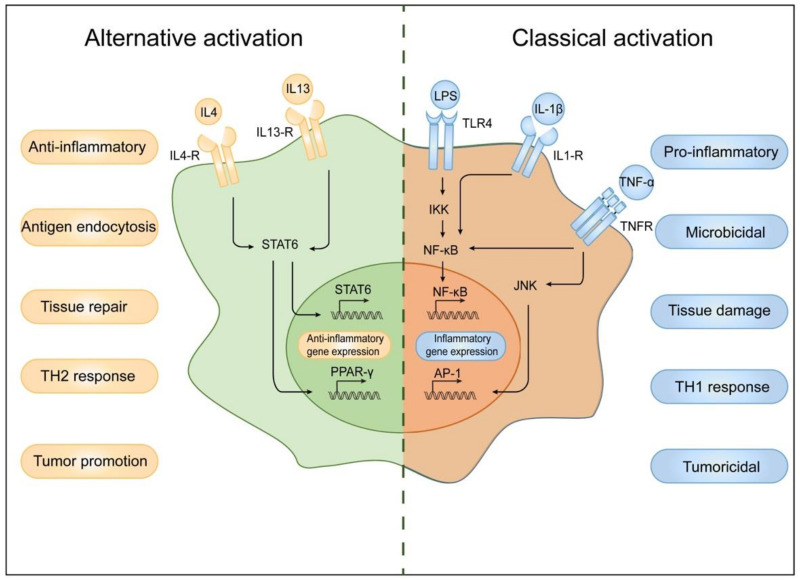
Cytokine stimulation leads to classical activation and alternative activation of macrophages. IL4 and IL13 activate macrophages to M2 polarization state through STAT6 and stimulate anti-inflammatory gene expression. M2 macrophages participate in Th2 response, anti-inflammatory, antigen endocytosis, and tumor promotion. On the other hand, when stimulated by LPS, IL-1β, and TNF-α, macrophages activate the inflammatory signaling cascades mediated by JNK and NF-κB, which stimulate M1 polarization of macrophages and inflammatory gene expression. M1 macrophages are involved in Th1 response, proinflammatory process, tissue damage, and so on.

In addition to cytokines secreted from or related to macrophages, macrophage autophagy is crucial for the regulation of systemic insulin sensitivity [194]. In macrophages, autophagy controls inflammation by regulating mitochondria turnover and ROS generation [194]. During inflammation and obesity, autophagy is downregulated in macrophages, and macrophage-specific autophagy knockout mice have impaired insulin sensitivity in liver and adipose tissues when fed an HFD, inhibiting ROS in macrophages with antioxidants can restore insulin sensitivity of adipocytes [195]. (Figure 2 and Figure 3)

## 5. Other Immune Cells and Insulin Resistance

In addition to macrophages, immune cells residing in adipose tissue and intestines, including cells of innate and adaptive immune systems, are also involved in chronic inflammation caused by obesity. In the process of obesity, immune cells, including Innate lymphoid type 1 cells (ILC1s), Innate lymphoid type 2 cells (ILC2s), eosinophils, mast cells, dendritic cells (DCs), neutrophils, T cells, and B cells, influence the polarization and recruitment of macrophages and play important roles in chronic inflammation and insulin resistance.

ILC2s and eosinophils participate in maintaining metabolic homeostasis states partially by maintaining the M2 polarization state of macrophages. ILC2s are a family of innate immune cells that mirror T cells [196]. They secrete Th2-associated cytokines, including IL-5 and IL-13, which promote the accumulation of M2 macrophages and eosinophils [197]. The deletion of ILC2s causes a significant decrease in VAT M2 macrophages and eosinophils and increases obesity and insulin resistance fed HFD [198]. Moreover, eosinophils have also been implicated in metabolic homeostasis and the maintenance of M2 macrophages [199]. Eosinophils content in adipose tissue is reduced in HFD-induced obesity and restored to lean levels when switching to a low-fat diet [200]. Eosinophils could produce IL-4 and IL-13, which promote differentiation of macrophages into the M2 state [198]. Impaired eosinophil accumulation in adipose tissue results in increased insulin resistance in obesity [198], and the absence of eosinophils leads to systemic insulin resistance [200].

Mast cells are first-line responders to invading pathogens by rapid degranulation ability [201], producing a wide range of inflammatory mediators, including histamines, prostaglandins, and proinflammatory cytokines (IFN-γ, TNF-α, IL-1β, IL-6), which trigger T-cell and M1 macrophage activation [202,203]. Liu et al. found that mast cells increased in obese adipose tissue, and mast cell deficiency can increase insulin sensitivity [204]. Additionally, some studies showed that DCs are involved in tissue recruitment and activation of macrophages; DC-deficient mice have a decreased number of ATMs and Kupffer cells, as well as improved glucose intolerance [205]. The transfer of bone-marrow-derived DCs to lean mice increases ATMs and Kupffer cells infiltration [205]. During obesity, neutrophils have high neutrophil elastase, neutrophil alkaline phosphatase, myeloperoxidase, IL-6, IL-1β, IL-12, IL-8, and TNF-α, and low expression of IL-10, which favors activation of M1 macrophages and development of chronic inflammation [206,207,208,209,210]. Neutrophil elastase impairs insulin signaling by promoting IRS-1 degradation, and elastase knockout in HFD mice improves glucose tolerance and increases insulin sensitivity [208]. Moreover, recent studies also found that ILC1s in the adipose tissue participate in developing inflammation and insulin resistance. HFD-induced obesity increases the number of ILC1s and induces them to produce IFN-γ and TNFα in the visceral adipose tissue (VAT), then ILC1s-derived IFN-γ and TNFα accelerate M1 macrophages accumulation and promote insulin resistance [211].

T cells, B cells, and macrophages are all in CLSs surrounding necrotic adipocytes [212]. Accumulation of macrophages and T cells within CLSs can predict the severity of obesity and insulin resistance [212]. T cells can be classified into CD4^+^ and CD8^+^ T cells by their phenotypic expression of the surface coreceptors. Increased recruitment and differentiation of CD8^+^ T cells have been found in both animal and human obese WAT [213]. T cell receptor beta chain-deficient mice improve obesity-induced hyperglycemia and insulin resistance by suppressing macrophage infiltration and reducing inflammatory cytokine expression [214]. The number of Th1 cells increases and produces more IFN-γ in obesity, which promotes M1 polarization of ATMs [215,216]. Adoptive transfer of Th1 cells into HFD lymphocyte-free mice results in increased inflammation, impaired glucose tolerance, and macrophage infiltration [214]. Moreover, Treg cells secrete IL10 in a lean state, preserving an anti-inflammatory environment and M2 macrophage polarization [217]. Treg cells in VAT decrease dramatically during obesity in HFD feeding mice [218]. Treg cell deficiency in adipose tissue increases the production of proinflammatory cytokines, M1 macrophage polarization, and insulin resistance [217]. In contrast with CD4^+^ T cells, CD8^+^ T cells are mainly considered cytotoxic. CD8^+^ T cells secrete perforins, granzymes, and cytokines that regulate the development and activation of macrophages [173]. Increased infiltration of CD8^+^ T cells in WAT was found in obese mice, which induces adipose tissue inflammation and attracts macrophage recruitment [219]. Depletion of CD8^+^ T cells could lower macrophage infiltration and adipose tissue inflammation and improve insulin resistance [219].

**Figure 4:**
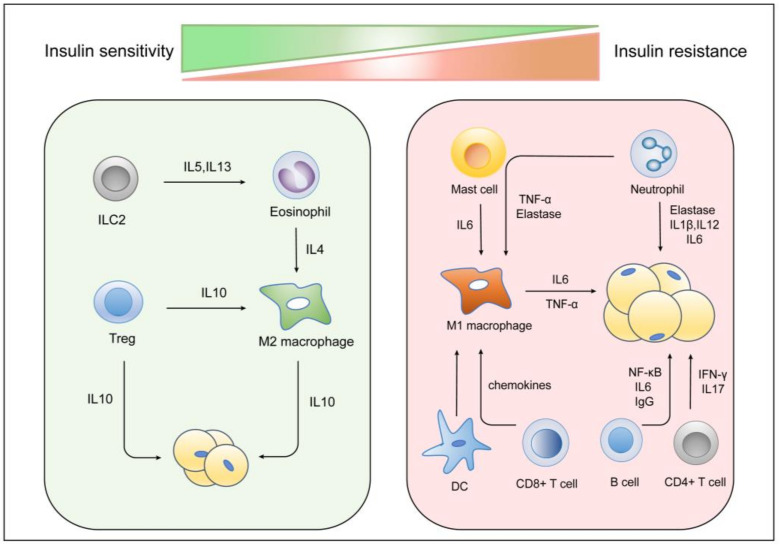
Role of the immune system in regulating polarization of macrophage and insulin resistance. In an insulin-sensitive state, ILC2 cells produce IL-5 and IL-13 to assist in the maturation and recruitment of eosinophils. Eosinophils and Treg cells promote the activation of M2 macrophages in the adipose tissue via IL-4 and IL-13 secretion. M2 macrophages and Treg cells secrete anti-inflammatory factors such as IL-10 to maintain insulin sensitivity in lean adipose tissue. In the obese state, recruitment of monocytes and differentiation into M1 macrophages are induced significantly. Mast cells produce IL-6 to trigger T-cell and M1 macrophage activation. Neutrophils contribute to M1 polarization of macrophages and impaired insulin signaling via the production of elastase, IL-6, and IL-1β. CD4^+^ and CD8^+^ T cells stimulate M1 macrophage polarization by secreting IFN-γ, IL17, and chemokines. B cells release NF-κB, IL6, and IgG that further contribute to M1 macrophage polarization and insulin resistance.

The impaired adaptive response of B cells leads to inflammation in obese people [220]. B cells secrete more IL-6 and NF-κB and less IL-10 in obese humans compared to lean individuals, which favors the recruitment of M1 macrophages [221]. Winer et al. demonstrated that B cells accumulate in VAT occurs early in HFD-fed mice and promote activation of T cells and M1 macrophages through the production of specific IgG antibodies, eventually inducing insulin resistance [222]. Obese B cell knockout mice have decreased systemic inflammation and improved insulin sensitivity and glucose tolerance compared to obese control mice [223] (Figure 4).

## 6. Macrophage-Related T2D Therapeutic Targets

Antidiabetic drugs act on adipose tissue and muscle to enhance insulin sensitivity and target the liver to inhibit glucose production, or stimulate β-cells to release insulin, thereby slowing the development of insulin resistance. However, they are not able to completely prevent or reverse the development of T2D, urgently necessitating new antidiabetic drugs [224]. Current studies used macrophage-derived factors or surface markers, including TNF-α, IL-1β, IL-6, miRNAs, and folate receptor (FR), as the therapeutic targets for diabetes, which have potential to be developed as pharmacological targets for the treatment of T2D [225,226].

TNF-α is identified as a potential target to treat insulin resistance in obesity; it is a classic proinflammatory cytokine and induces M1 polarization of macrophages. Treatment with shRNA to downregulate TNF-α contributes to improving IRS-1 phosphorylation and insulin response, which improves insulin sensitivity [227]. Burska et al. concluded that TNF-α inhibitor treatment improves insulin sensitivity in rheumatoid arthritis (RA) patients [228]. The topical addition of anti-TNF-α neutralizing antibodies could shift the macrophages towards M2 phenotype and accelerate wound healing [229]. Wang et al. reported a glucose-sensitive TNFα-antibody-delivery system for controlling local long-term inflammation and improving osteogenesis in T2D [230]. The clinical trials of anti-TNF-α antibodies Ro 45-2081 and CDP571 did not show any improvement in glucose homeostasis and insulin sensitivity [231,232]. Relevant studies are needed to prove the effectiveness of TNF-α target. In addition, when IKK/NF-κB pathway is inhibited by pharmacological inhibitors of IKK2 salicylates and aspirin, the animal models show improved obesity-induced insulin resistance and reduced TNF-α production [173].

IL-1β is elevated in obesity and induces insulin resistance by inhibiting the insulin signaling pathway [225,233]. IL-1β antibody treatment improves insulin resistance, glycemic control, and β-cell functions in mice with HFD-induced obesity [225]. IL-1R antagonist Anakinra also improves the glycemia β-cell functions and reduces systemic inflammation in patients with T2D; thus, it is approved for treating patients with RA (ClinicalTrials.gov identifier: NCT00303394) [234]. Human mAbs canakinumab and Xoma 052 are used to neutralize IL-1β specifically and support that blockade of IL-1β restores the regeneration of β-cells [235]. Canakinumab is tested in high-risk patients with T2D (ClinicalTrials.gov identifier: NCT01327846) and shows modest improvement in HbA1c, glycemia, and insulinemia [158]. Additional results of ongoing clinical trials are needed to clarify the function of IL-1β target.

IL-6 is another proinflammatory cytokine that participates in the development of T2D. Human monoclonal antibody Tocilizumab could inhibit IL-6 signaling selectively, and treatment with Tocilizumab causes a significant reduction of HOMA-IR and insulin resistance in non-diabetic patients with RA [236]. These studies suggest that inhibiting IL6 may improve insulin resistance and T2D. Further, IL-10 is an anti-inflammatory factor, which induces M2 activation of macrophages and improves obesity-induced insulin resistance [119]. Recombinant IL-10 has been proved safe in clinical trials to treat autoimmune diseases, neurodegenerative disorders, and so on [237]. Thus IL-10 may also be a promising target for treating T2D.

Macrophages could secrete miRNAs encapsulated in extracellular vesicles to exert anti-inflammatory or proinflammatory effects, and miRNAs may also contribute to the development of insulin resistance by regulating macrophage polarization. Since miRNAs are endogenous regulators whose levels are often deregulated in T2D, the therapeutic goal is to restore miRNAs to normal levels [226]. Presently, there are two main therapeutic methods: miRNA mimics to restore the miRNAs downregulated in diabetes, miRNA mimics that are synthetic RNA duplexes, liposomes, and adeno-associated virus preparations designed to package miRNAs [238,239]; the other method is to inhibit miRNAs that express significantly higher than normal, locked nucleic acid anti-miRNAs and morpholinos that are efficient inhibitors of miRNA decrease the plasma cholesterol with very low toxicity in mice [240,241]. Due to the instability of miRNA mimics and the difficulty in specific targeting, miRNA therapy needs to be developed further to treat T2D.

Moreover, therapeutic drugs with folate conjugates could selectively impair FR active macrophages [242]. FR is a marker of macrophage activation. While nonactivated resident macrophages do not express FR, other cells, including granulocytes, lymphocytes, or erythrocyte-enriched populations, show poor folate-conjugate binding. Furthermore, folate-FITC binding to active macrophages is FR-specific, which makes the folate receptor an optimal target for active macrophages involved in insulin resistance [243].

## 7. Conclusions

Accumulating evidence showed that macrophages play central roles in chronic inflammation and insulin resistance induced by obesity. Macrophages participate in inflammatory pathways including JNK, IKK/NF-κB, and JAK/STAT through secreting inflammatory factors, such as TNF-α, IL-6, IL-1β, and LTB4, thereby inducing chronic inflammation of adipose tissue, liver, and muscle, further leading to insulin resistance. Other immune cells, including ILC2s, eosinophils, mast cells, DCs, neutrophils, T cells, and B cells, affect the polarization state of macrophages, which in turn participate in the induction of insulin resistance and T2D. In recent years, there have been many discoveries in the field of immune metabolism, and direct or indirect targeting of macrophage-secreted factors or macrophage-related markers can improve insulin resistance and reduce the development of T2D by mechanisms including enhancing insulin sensitivity, reducing oxidative stress, and reducing inflammation et al. Current research hotspots include targeted therapy of TNF-α, IL-1β, IL-6, and miRNAs. Although many problems remain unsolved, macrophages may be considered as promising targets for the treatment of T2D.
